# Effect of Cast Defects on the Corrosion Behavior and Mechanism of UNS C95810 Alloy in Artificial Seawater

**DOI:** 10.3390/ma13071790

**Published:** 2020-04-10

**Authors:** Xu Zhao, Yuhong Qi, Jintao Wang, Zhanping Zhang, Jing Zhu, Linlin Quan, Dachuan He

**Affiliations:** 1 Department of Materials Science and Engineering, Dalian Maritime University, Dalian 116026, China; zx1988@dlmu.edu.cn (X.Z.); 18342299941@163.com (J.W.); zzp@dlmu.edu.cn (Z.Z.); 2 Dalian Marine Propeller Co., Ltd., Dalian 116021, China; jingzhu2009@yeah.net (J.Z.); quanlinlin@163.com (L.Q.); xyz@dmpp.cn (D.H.)

**Keywords:** nickel-aluminum bronze, cast defects, electrochemical corrosion, mechanism, microstructure

## Abstract

To study the effect of cast defects on the corrosion behavior and mechanism of the UNS C95810 alloy in seawater, an investigation was conducted by weight loss determination, scanning electron microscopy (SEM), confocal laser scanning microscopy (CLSM), X-ray diffraction (XRD) and electrochemical testing of the specimen with and without cast defects on the surface. The results show that the corrosion rate of the alloy with cast defects is higher than that of the alloy without cast defects, but the defects do not change the composition of the resulting corrosion products. The defects increase the complexity of the alloy microstructure and the tendency toward galvanic corrosion, which reduce the corrosion potential from −3.83 to −86.31 mV and increase the corrosion current density from 0.228 to 0.23 μA⋅cm^−2^.

## 1. Introduction

Due to its good mechanical properties, corrosion fatigue resistance, corrosion resistance and cavitation resistance, Ni-Al bronze (NAB) has been widely used for marine components, such as ship propellers, pumps and valves [1,2,3]. NAB parts used in seawater are inevitably subject to corrosion by the seawater. To date, there are many studies on NAB corrosion behavior in seawater [1,3,4,5,6,7,8]. The results showed that an α phase corroded while a κ phase did not corrode in near-neutral seawater and 3.5% NaCl solution [1,8,9]. Neodo et al. [7] studied the corrosion behavior of NAB in a 3.5% NaCl solution with different pH values. It was found that the κ phase is preferentially corroded when the pH<4 but the α phase dissolves first when the pH > 4. The results of Weill-Couly et al. [10] showed that the corrosion resistance of NAB in seawater was related to its microstructures. The phase transformation of a small amount of β’→ α + κ could effectively improve the corrosion resistance of the alloy in seawater. The excellent corrosion resistance of NAB is derived from a corrosion product film produced on the surface [4,11,12,13,14]. Schüssler et al. [4] proposed that the corrosion product film produced on the surface of NAB contained an inner layer of Al_2_O_3_ and an outer layer of Cu_2_O; furthermore, a thickness of 800–1000 nm could reduce the corrosion rate by 20–30 times. If time is sufficient, corrosion products such as Cu_2_(OH)_3_Cl will also form on the outer layer of the film [12,13].

The as-cast NAB pieces used for ship propellers are very large, weighing as much as 200 t. Therefore, cast defects are unavoidable. Large and concentrated defects can be repaired in various ways to reduce the adverse effects of defects. For example, Sabbaghzadeh et al. [15] repaired NAB by fusion welding, and the galvanic corrosion current between the welded and the as-cast microstructures was only a few nA. Hanke et al. [16] applied a friction surface treatment for as-cast NAB, resulting in refinement and homogenization of the surface microstructure, which repaired cast defects. Ni et al. [17] used friction stir processing for as-cast Ni-Al bronze and compared the weight loss of the treated NAB and as-cast NAB in 3.5% NaCl solution. It was found that the weight loss of the treated NAB was significantly smaller than that of as-cast NAB, improving corrosion performance. That is, welding can improve the corrosion resistance of copper alloys with large cast defects in seawater.

However, for small and dispersed defects, they are not only unavoidable but also difficult to repair. Therefore, NAB propellers that are put into use generally have small cast defects, and their effect on the corrosion behavior of NAB in practical applications should be considered. Unfortunately, there is little literature available on this issue. Therefore, the effect of small cast defects on the corrosion behavior of the UNS C95810 alloy will be investigated in this paper by comparing the corrosion rate, morphology, product composition and electrochemical corrosion parameter of the specimen with and without defects.

## 2. Experiment

### 2.1. Materials and Corrosive Medium

The experimental material was UNS C95810 and its chemical composition is shown in Table 1. 

The measured yield strength, tensile strength and elongation were 266 MPa, 679 MPa and 29.5%, respectively. Specimens with and without defects were taken from a UNS C95810 on a large as-cast sample, and they were cut out (of 10 × 10 × 10 mm^3^) and observed under optical microscopy to determine whether there was a defect on the surface of each sample. For simplicity, the specimens with and without defects are represented by S-Defect and S-Cast, respectively. To effectively control the uncertainties of the test, artificial seawater was used as a corrosive medium, and its main chemical composition from ASTM D1141 was listed in Table 2.

### 2.2. Preparation of Test Specimens

#### 2.2.1. Specimens for Electrochemical Tests

Each of the specimens was mounted in plastic tubes by a two-component epoxy resin (WSR618, Nantong Xingchen Synthetic Material Co., Ltd, Nantong, China) with a Cu wire welded on their back, leaving an area of 0.38 cm^2^ to be in contact with the artificial seawater. The study surface of each specimen was ground to 1000 grit by silicon carbide abrasive paper and polished. Then, the sample was cleaned with absolute ethanol to remove organics, embedded polishing media, etc.

#### 2.2.2. Specimens for the Corrosion Test and Microstructure Analysis

Each specimen used for the weight loss measurements, scanning electron microscopy (SEM, Supra-55-sapphire, Carl Zeiss AG, Jena, Germany), confocal laser scanning microscopy (CLSM, OLS4000, Olympus, Tokyo, Japan) and X-ray diffraction (XRD, D/MAX-Ultima+, Rigaku, Tokyo, Japan) was mounted in plastic tubes by a two-component epoxy resin, leaving an area of 1 cm^2^ to be in contact with the artificial seawater. The test surface of each specimen was ground to 1000 grit by silicon carbide abrasive paper and polished. Then, the sample was cleaned with absolute ethanol to remove organics, embedded polishing media, etc.

### 2.3. Measurement and Characterization

#### 2.3.1. Weight Loss

The specimens prepared in 2.2.2 were ultrasonically cleaned in ethanol, dried with blowing air and weighed as m_1_. They were then immersed in artificial seawater. After immersion for 72 h, 168 h, 432 h, 720 h and 960 h, the specimens were removed, rinsed with deionized water, dried, and weighed as m_2_. Subsequently, they were immersed in HCl solution (containing 500 mL of commercially available 32% HCl and 500 mL of deionized water) for 2 min to remove the corrosion products and weighed as m_3_ after ultrasonically cleaning and drying. The weight loss of a fresh sample after immersion in this solution for 2 min was less than 0.1 mg to ensure the base metal was not attacked vigorously by the acid. The weight loss of the specimen and the weight of the corrosion products were noted as m_1_–m_3_ and m_2_–m_3_, respectively. The weight loss rate = $\frac{m_{1}-m_{3}}{S\cdot t}$, where *S* is the surface area of the sample in contact with artificial seawater and *t* is the immersion time.

#### 2.3.2. Microstructure Analysis and Corrosion Morphology Observation

The surface morphology of the specimen prepared in as described in Section 2.2.2 was observed by SEM, and the defects and their surrounding as-cast microstructures were analyzed by an energy dispersive spectrometer (EDS, Ultim Extreme, OXFORD Instruments, Oxford, UK) before and after immersion in artificial seawater. After immersion for 72 h and 240 h, they were removed, rinsed with deionized water, dried, and observed by CLSM. After immersion in artificial seawater for 720 h, the washed and dried specimens were subjected to SEM observation and EDS analysis.

#### 2.3.3. XRD

The specimens prepared in 2.2.2 were tested using an X-ray diffractometer (D/MAX-Ultima^+^, Rigaku, Tokyo, Japan) with Co Kα radiation. The diffraction angle range was from 10° to 100°. The specimens that had been tested by XRD were immersed in artificial seawater. After immersion for 240 h and 720 h, they were removed, rinsed with deionized water, dried, and tested by XRD.

#### 2.3.4. Electrochemical Test

Electrochemical measurements were conducted in a typical three-electrode system. The working electrode was the surface of the specimen with an area of 0.38 cm^2^ prepared in 2.2.1. A platinum net and a saturated calomel electrode served as the counter and reference electrode, respectively. The test medium was 100 mL of artificial seawater. The polarization curves were recorded at a sweep rate of 1 mV/s from -500 mV to 500 mV vs. the open circuit potential using an IM6ex Electrochemical Workstation (IM6ex, ZAHNER, Kronach, Germany). The voltage perturbation was 10 mV. To avoid any inconsistency of the experiment, two or three repeated tests were carried out for each sample. The electrochemical corrosion parameters in this paper were then obtained by fitting a linear polarization region of approximately 10 mV around the resting potential [19].

## 3. Result

### 3.1. Weight Loss

**Figure 1:**
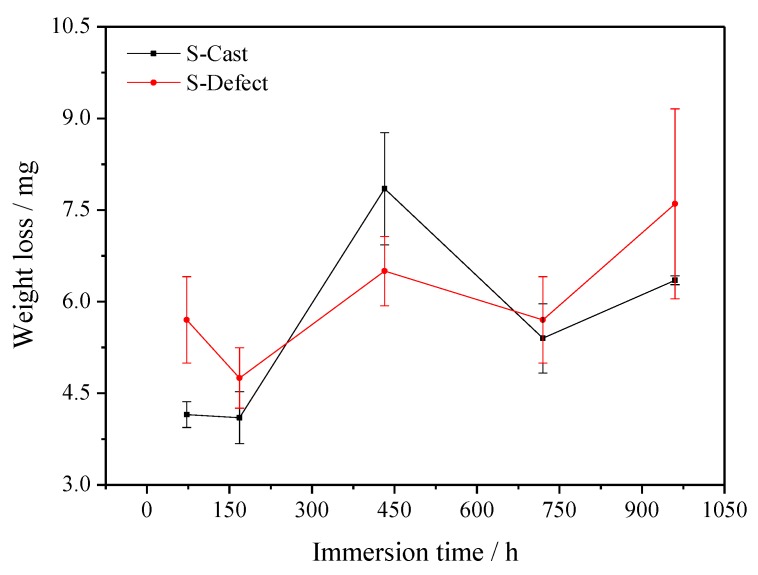
Weight loss of S-Cast and S-Defect immersed for different time periods in artificial seawater.

**Figure 2:**
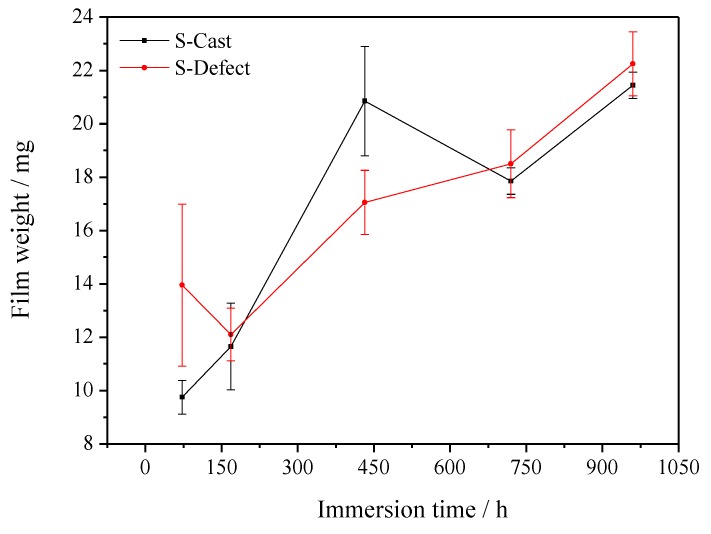
Film weight of S-Cast and S-Defect immersed for different time periods in artificial seawater.

**Figure 3:**
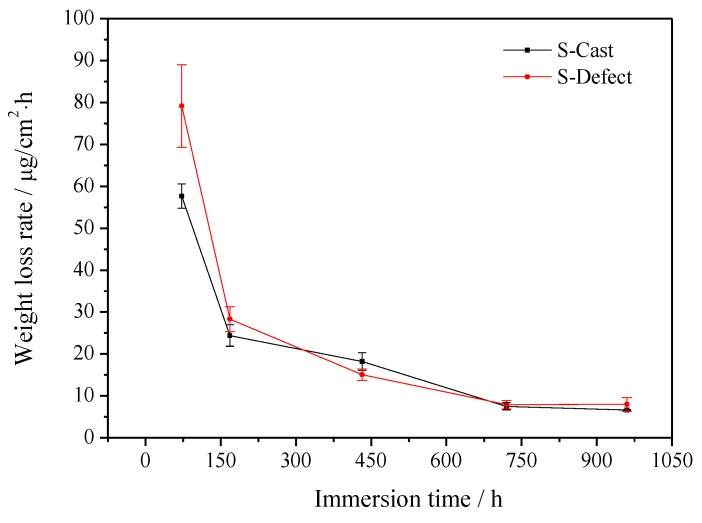
Weight loss rate of S-Cast and S-Defect immersed for different time periods in artificial seawater.

The test results of S-Cast and S-Defect weight loss in artificial seawater are shown in Figure 1, Figure 2 and Figure 3. The results show that the weight loss and film weight of S-Cast and S-Defect generally show a gradual increase trend with corrosion time but their weight loss rates gradually decrease with corrosion time. Moreover, the weight loss, film weight and weight loss rate of S-Defect are larger than that of S-Cast. Therefore, the defects accelerate the corrosion of UNS C95810. Defects induce a discontinuous corrosion product film on NAB in seawater, which results in a decrease in the protective effect of the film on the substrate and an increase in the corrosion rate and the thickness of the film.

### 3.2. Microstructure and Corrosion Morphology

**Figure 4:**
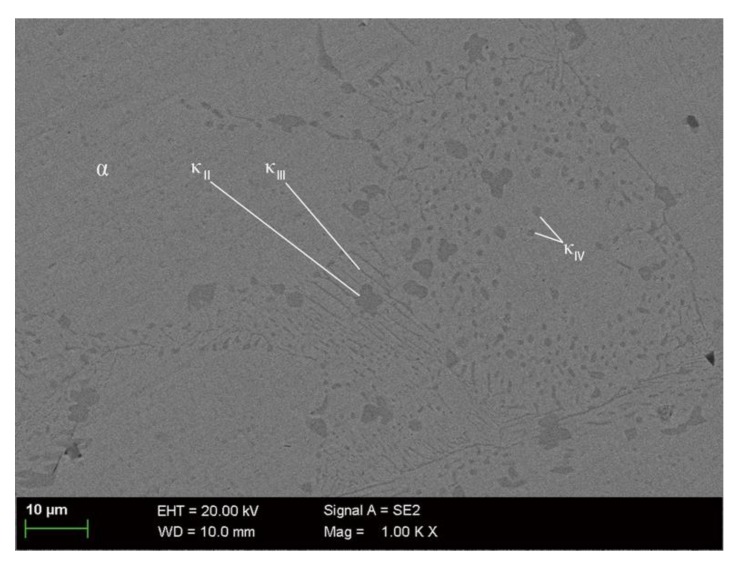
The microstructure of S-Cast before corrosion.

The normal microstructure of UNS C95810 is mainly composed of a α phase matrix and a dispersed κ phase (Figure 4).

**Figure 5:**
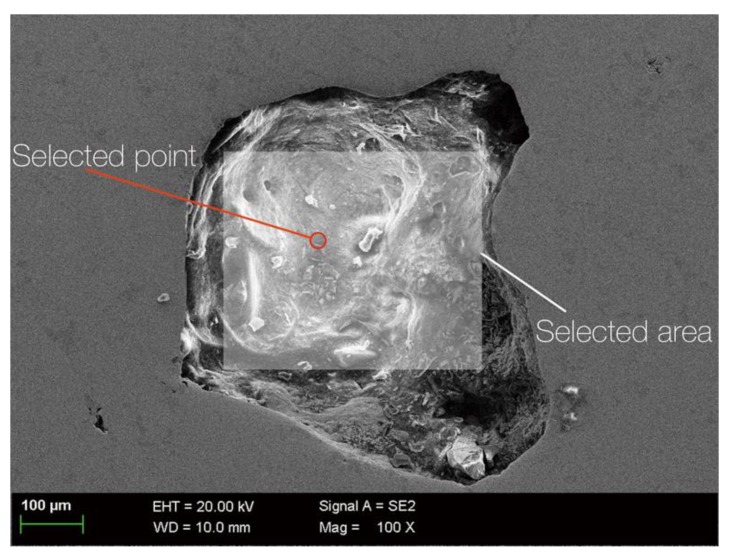
Surface and point scan of S-Defect before corrosion.

According to the composition and shape of the κ phase, it can be divided into κ_II_, κ_III_ and κ_IV_. The phase κ_II_ is an intermetallic compound based on Fe_3_Al, which is flower-like or spherical with a size of 5 to 10 μm and distributed at the boundary of the α phase [9]. The κ_III_ phase is a lamellar intermetallic compound based on NiAl [9]. The κ_IV_ phase is an intermetallic compound based on Fe_3_Al and is distributed in the α phase with a size smaller than 2 μm [9]. The size of the α phase is generally more than 100 μm [20]. To determine the chemical composition of the defects, EDS tests of selected area and selected points (Figure 5) are carried out. The results show that there is only copper in selected point while there are many oxygen and few chlorine elements in alloys in selected areas, as shown in Table 3. 

It can be inferred that the microstructures in defects mainly include pure copper, an oxide of copper and aluminum and the κ phase.

**Figure 6:**
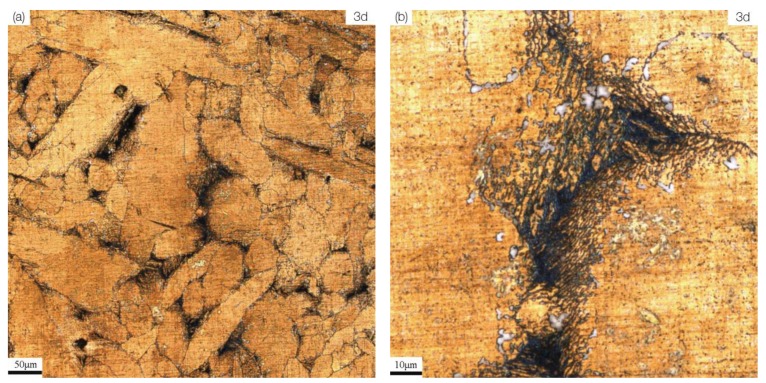

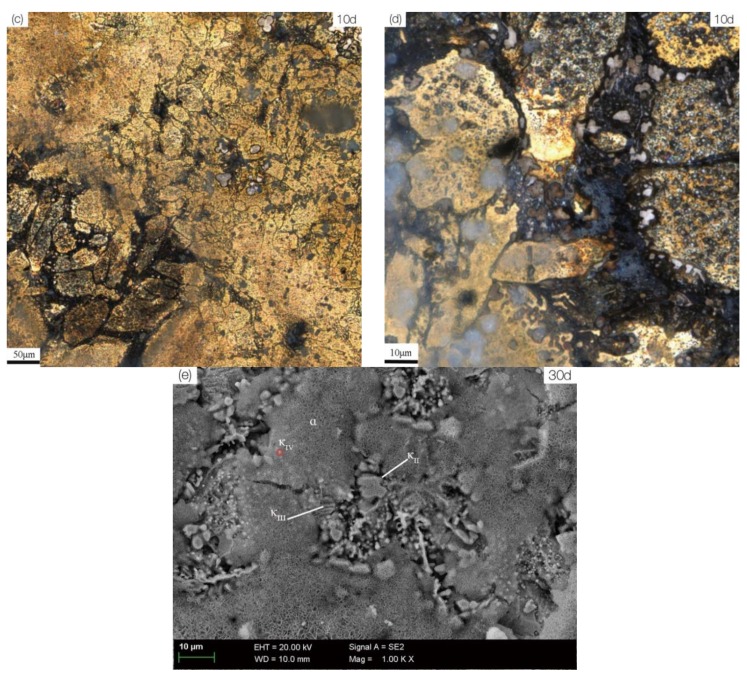
The corrosion morphology of for S-Cast immersed in artificial seawater at different times: (**a**) 3 d; (**b**) local magnification at 3 d; (**c**) 10 d; (**d**) local magnification at 10d; (**e**) 30 d.

S-Cast and S-Defect differ in their corrosion processes. S-Cast is preferentially corroded at the boundary of the αphase and some locations within the α phase, and the corrosion near the boundary of the α phase is more serious (Figure 6a,b). 

**Figure 7:**
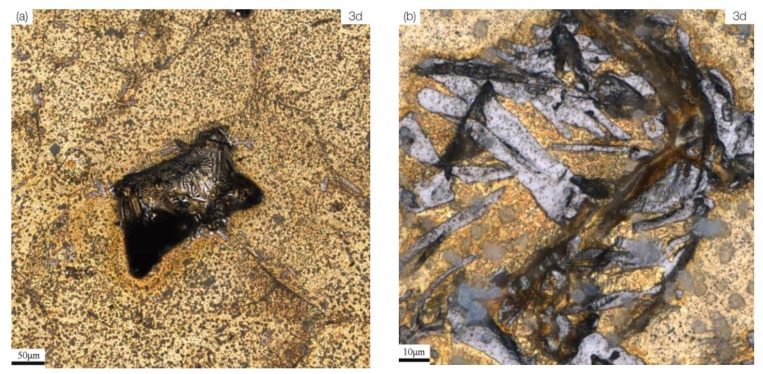

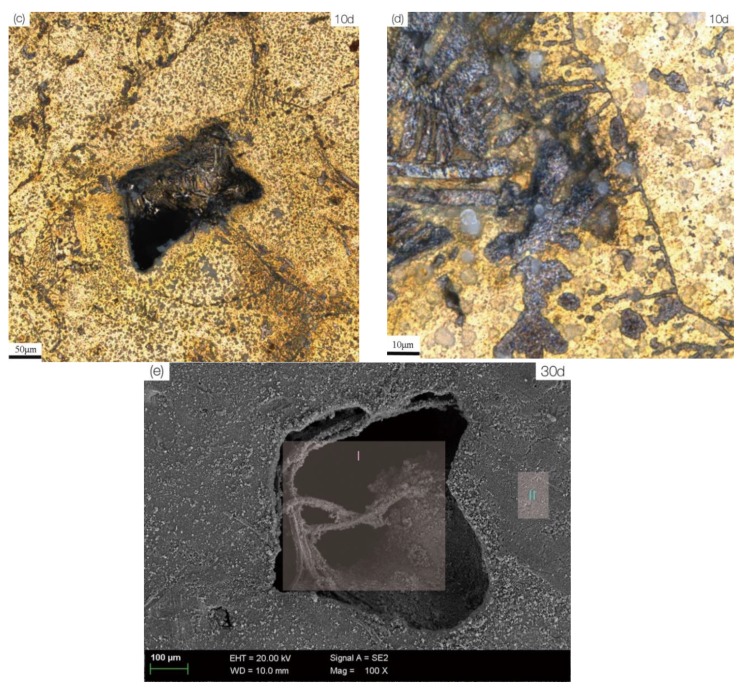
The corrosion morphology of for S-Defect immersed in artificial seawater at different times: (**a**) 3 d; (**b**) local magnification at 3 d; (**c**) 10 d; (**d**) local magnification at 10d; (**e**) 30 d.

As the corrosion progresses, the α phase also begins to undergo significant corrosion (Figure 6c,d). When the corrosion reaches 30 days, obvious corrosion products have formed on the surface of the alloy (Figure 6e). S-Defect is preferentially corroded at the defects, and the corrosion near the defects is more serious (Figure 7a,b). 

After the corrosion occurs at the defects and their vicinity, the corrosion at the αphase boundary and some locations within the αphase gradually becomes apparent (Figure 7a–d). When the corrosion reaches 30 days, a large area of severe corrosion is occurred at the defects compared to other locations (Figure 7e). In addition to affecting the location of preferential corrosion, defects increase the complexity of UNS C95810 microstructures, leading to an increase in tendency toward galvanic corrosion, thus accelerating the corrosion of UNS C95810 (Figure 6 vs. Figure 7). To determine whether there is a difference between the corrosion product composition of the defects and that of the surrounding as-cast microstructures, EDS analysis is performed on the position shown in Figure 7e. The results (Table 4) show that there is almost no composition difference between the corrosion product of the defects and that of the surrounding as-cast microstructures.

### 3.3. Corrosion Product Film

**Figure 8:**
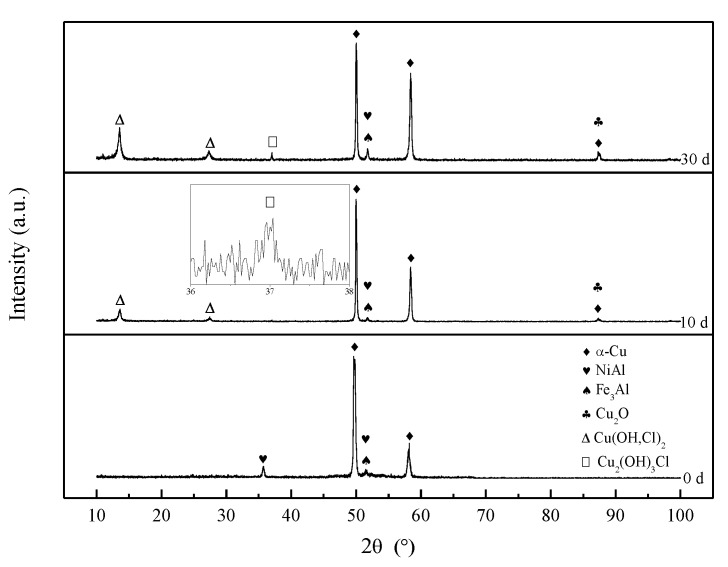
The XRD patterns of S-Defect immersed for different time periods in artificial seawater.

**Figure 9:**
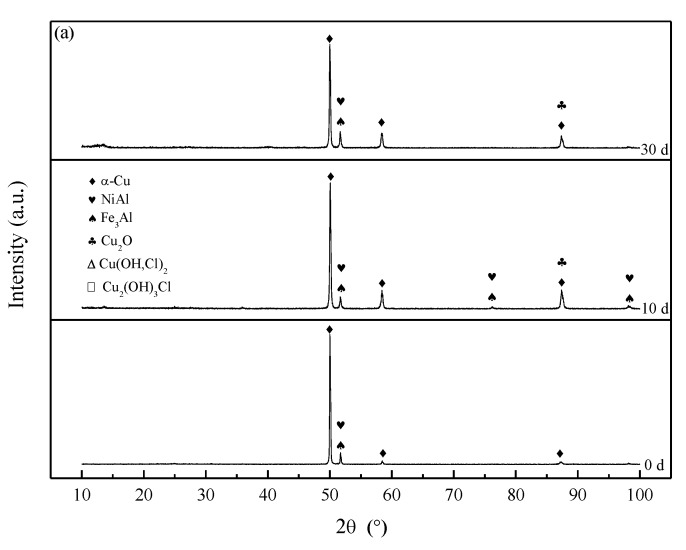

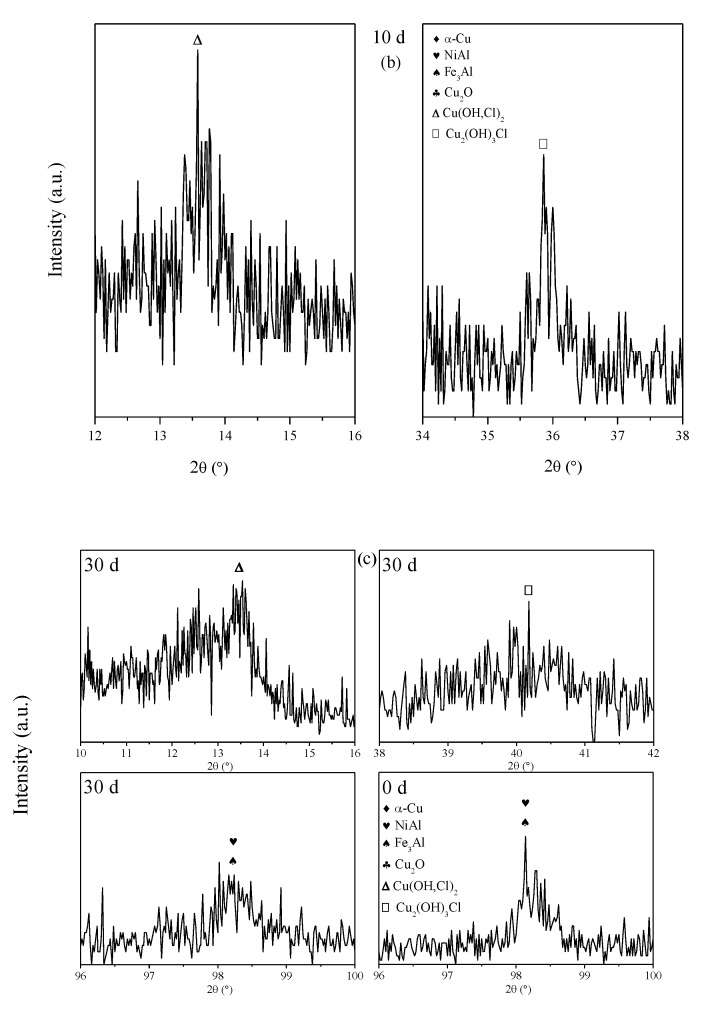
The XRD patterns of (**a**) S-Cast immersed for different time periods in artificial seawater and (**b**,**c**) S-Cast partial magnification.

The XRD results of S-Defect and S-Cast for different immersion periods are shown in Figure 8 and Figure 9. Cu_2_O, Cu(OH,Cl)_2_ and Cu_2_(OH)_3_Cl are detected in the XRD results of S-Defect and S-Cast. Cu_2_O changes little with corrosion while Cu(OH,Cl)_2_ and Cu_2_(OH)_3_Cl vary greatly with corrosion (Figure 8). Cu(OH,Cl)_2_ is more abundant in the corrosion products after 10 days than that of Cu_2_(OH)_3_Cl (Figure 8). Therefore, it is considered that during the corrosion process, Cu dissolves to Cu^+^, and then it reacts with Cl^-^ to form CuCl_2_^−^ [13,20].

Over time, it converts to Cu_2_O [20,21]. As the amount of Cu_2_O increases, it will react with Cl^−^ to form more stable phases, such as Cu_2_(OH)_3_ Cl [20,21,22]. This is similar to the experimental results of Song [21] and Du [22]. Song [21] proposed that Cu_2_(OH)_3_Cl was located on the outermost layer of a NAB corrosion product film. Du [22] proposed that Cu(OH)_2_ and CuCl_2_ were located between Cu_2_O and Cu_2_(OH)_3_Cl. It is believed that since Al is more active than Cu, it first dissolves to form Al_2_O_3_, and then Cu dissolves to form CuO and Cu_2_O. Cu(OH)_2_, CuCl_2_ and Cu_2_(OH)_3_Cl are formed on the basis of products formed by the dissolution of Cu. Therefore, they should be located on the outer layers of the Al_2_O_3_, CuO and Cu_2_O. The XRD results in this study indicate that the Al_2_O_3_, Cu_2_O, CuO, Cu(OH)_2_, CuCl_2_ and Cu_2_(OH)_3_Cl phases were detected on the corrosion product film with increasing corrosive time. In summary, the corrosion product film of UNS C95810 has a three-layer structure.

### 3.4. Electrochemistry

**Figure 10:**
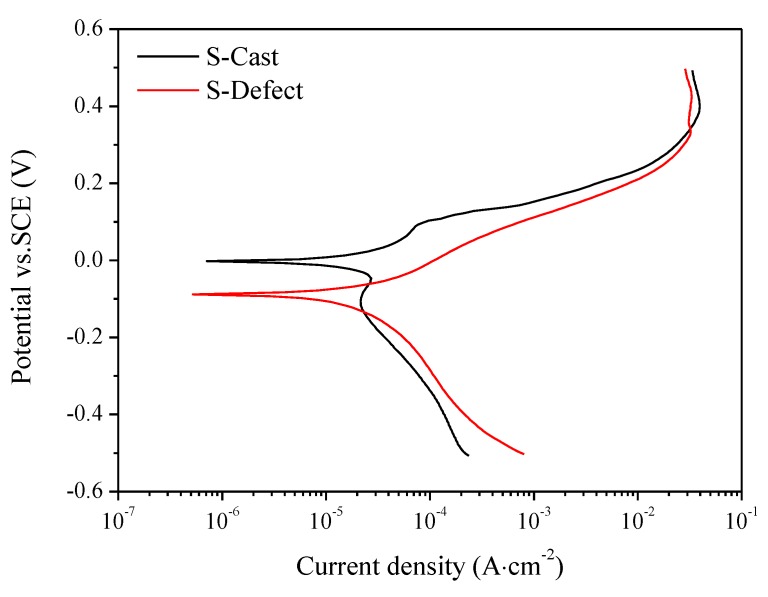
The polarization curve of S-Cast and S-Defect.

The polarization curves of S-Cast and S-Defect in artificial seawater are shown in Figure 10. 

The electrochemical corrosion parameters obtained by fitting a linear polarization region of approximately 10 mV around the resting potential are shown in Table 5. 

The corrosion potential and corrosion current density of S-Defect are −86.31 mV and 0.23 μA⋅cm^−2^, respectively, while those of S-Cast are −3.83 mV and 0.228 μA⋅cm^-2^, respectively. Generally, defects decrease the corrosion potential and increase the corrosion current density. Defects increase the tendency toward galvanic corrosion of UNS C95810, which leads to the promotion of the corrosion driving force, thus accelerating corrosion. Therefore, S-Defect has a lower corrosion potential and a larger corrosion current density than those of S-Cast.

## 4. Discussion

### 4.1. Corrosion Rate of UNS C95810

**Figure 11:**
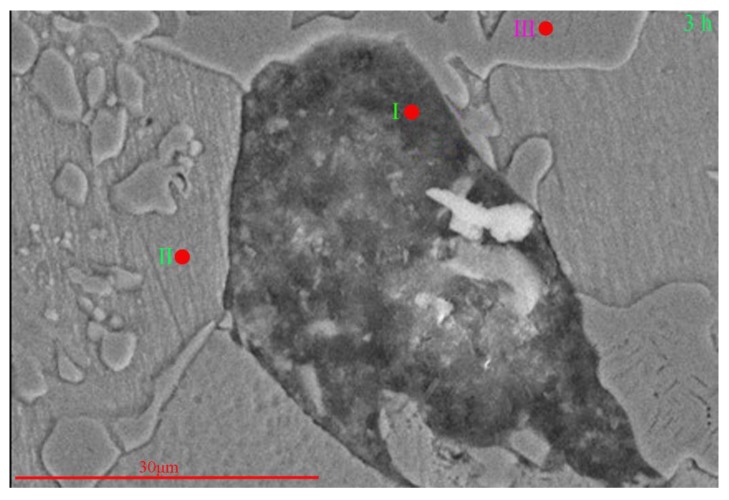
SEM image of S-Defect immersed in artificial seawater for 3 h.

Defects can affect the diffusion of Cl^−^ and the corrosion driving force, thus accelerating the corrosion of NAB. The presence of defects causes the passivation film formed on NAB to become uneven. Generally, the electric field strength in the passivation film (*ε*) obeys the following formula: $\varepsilon =\frac{V}{l}$, where *V* is the potential difference between the two sides of the passivation film and *l* is the thickness of the passivation film [19]. Because the passivation film at the defects is thinner than that at other locations [19], the field strength at the defects is higher than that at other locations. This causes Cl^-^ to diffuse more easily to the defects and to enrich there (Figure 11 and Table 6), resulting in faster destruction of the passivation film. 

Once the passivation film is broken, the substrate is directly in contact with the corrosive medium, which accelerates the corrosion rate. Therefore, the presence of defects affects the diffusion of Cl^−^ and the corrosion process.

### 4.2. Corrosion Product Film Structure of S-Defect

The results of the XRD test show that the X-ray diffraction pattern of both the S-Cast and S-Defect samples are basically the same, as shown in Figure 8 and Figure 9, but the intensity of some phases in the corrosion products is different. Therefore, they have similar corrosion product film structures but different corrosion product film thicknesses (Figure 2, Figure 8 and Figure 9). It had been reported that a corrosion product film with Cu_2_O in the outer layer and Al_2_O_3_ in the inner layer was formed on a NAB [4] and a ternary Cu-Al-Ni alloy [23,24]. Cu_2_O and Al_2_O_3_ were formed by the following reactions [12,13,22,24,25]:

(1) $$\mathrm{Al}\left(s\right)+4{\mathrm{Cl}}^{-}\to {\mathrm{AlCl}}_{4}{}^{-}+3e^{-}$$

(2) $${\mathrm{AlCl}}_{4}{}^{-}+2H_{2}O\to {\mathrm{Al}}_{2}O_{3}\left(s\right)+4{\mathrm{Cl}}^{-}+3H^{+}$$

(3) $$\mathrm{Cu}\left(s\right)\to {\mathrm{Cu}}^{+}+e^{-}$$

(4) $${\mathrm{Cu}}^{+}+{\mathrm{Cl}}^{-}\to \mathrm{CuCl}\left(s\right)$$

(5) $$\mathrm{CuCl}\left(s\right)+{\mathrm{Cl}}^{-}\to {\mathrm{CuCl}}_{2}{}^{-}$$

(6) $$2{\mathrm{CuCl}}_{2}{}^{-}+H_{2}O\to {\mathrm{Cu}}_{2}O\left(s\right)+4{\mathrm{Cl}}^{-}+2H^{+}$$

Cu_2_O is a p-type semiconductor with cation vacancies. It can accept foreign ions, such as Ni and Fe ions, to incorporate into inner cation vacancies, further improving the protection from the film [14,26,27]. Based on previous research [4,14,21,23,24,26,27], Cu_2_O should be the main corrosion product in the inner layer of the film for both S-Cast and S-Defect. Fe and Ni is enriched in the inner layer by incorporating into the lattice of Cu_2_O. The microstructures of the S-Cast and S-Defect are complex, resulting in different Cu dissolution rates in different phases. Because Al_2_O_3_ cannot form simultaneously on different phases, the discontinuous Al_2_O_3_ layer on the surface is not impermeable to the passage of cuprous cations [20]. As a result, the inner layer of the corrosion product film consists of Al_2_O_3_ and Cu_2_O with the incorporation of Fe and Ni [20]. In the XRD tests, Cu(OH,Cl)_2_ and Cu_2_(OH)_3_Cl are detected in the corrosion products of S-Cast and S-Defect. They could form by the following reactions [13,25,26,28]:

(7) $${\mathrm{Cu}}^{+}+\frac{1}{2}O_{2}+e^{-}\to \mathrm{CuO}\left(s\right)$$

(8) $$\mathrm{CuO}\left(s\right)+H_{2}O\to \mathrm{Cu}{\left(\mathrm{OH}\right)}_{2}\left(s\right)$$

(9) $${\mathrm{Cu}}_{2}O\left(s\right)+{\mathrm{Cl}}^{-}+2H_{2}O\to {\mathrm{Cu}}_{2}{\left(\mathrm{OH}\right)}_{3}\mathrm{Cl}\left(s\right)+H^{+}+2e^{-}$$

**Figure 12:**
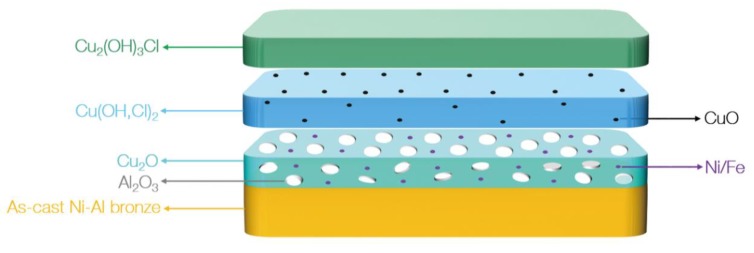
The corrosion product film structure of S-Defect immersed in artificial seawater for a long time.

According to Song’s research [21] on a NAB corrosion product film, Cu_2_(OH)_3_Cl was located in the outer layer of the NAB corrosion product film. In the structure of the NAB corrosion product film proposed by Du [22], Cu(OH)_2_ or CuO was located between Cu_2_O and Cu_2_(OH)_3_Cl. Therefore, combined with the above analysis and the results of the XRD test, it can be concluded that the structure of the S-Defect corrosion product film is as follows (Figure 12): the inner layer is Al_2_O_3_ and Cu_2_O with the incorporation of Fe and Ni, the middle layer is Cu(OH,Cl)_2_ or CuO, and the outer layer is Cu_2_(OH)_3_Cl.

### 4.3. Microstructure and Corrosion Behavior of UNS C95810

The stability of the phase and its corrosion products can affect the corrosion behavior, and the stability of the phase can be expressed with a work function. There is a corresponding relationship between work function and Volta potential: $V_{CPD}=\left(\Phi_{tip}-\Phi_{sample}\right)∕e$*,* where $V_{CPD}$ is the Volta potential, $\Phi_{tip}$ and $\Phi_{sample}$ are the work functions of the tip and the sample, respectively, and *e* is the value of the electronic charge [29]. The value of $\Phi_{tip}$ is constant. Thus, higher $V_{CPD}$ results in lower $\Phi_{sample}$. The work function is defined as the minimum energy required for an electron to escape from the surface of a solid. A lower work function of a material represents more likely occurrences of corrosion [30]. Therefore, a substance with a higher Volta potential is more susceptible to corrosion. From the measurements of the Volta potential of an as-cast NAB by Song, it can be seen that the Volta potential of the κ_IV_ phase was higher than that of the α phase but lower than that of the κ_II_ phase [21]. Hence, the stability of the phases from low to high should be κ_II_, κ_IV_ and α. However, in near-neutral artificial seawater, Al_2_O_3_ is formed on the surface of the Al-rich κ phase and its stability is much higher than that of Cu_2_O, so the κ phase as the cathode phase will not continue to corrode but accelerates the corrosion rate of the surrounding microstructures [31]. Since the κ_IV_ phase and κ_II_ phase are located in the α phase and the boundary of the α phase, corrosion preferentially occurs in the αphase boundary and some locations in the α phase. The Volta potential between the κ_II_ phase and α phase is 60–80 mV, while that between the κ_IV_ phase and α phase is 20–40 mV [21]. The areas with a higher Volta potential have a stronger driving force for corrosion. Therefore, the corrosion in the α phase boundary is more prone to occur than that in the α phase.

The existence of defects accelerates the corrosion process to a certain extent, mainly due to the following reasons. First, the defect microstructures mainly include oxides of copper and aluminum, along with pure copper. This is a more complex phase composition than the as-cast microstructures. Such a phase composition makes it easier for the inside of defects and between the defects and the surrounding microstructures to form a galvanic corrosion, thereby accelerating the corrosion of the defects and the microstructures near the defects. Second, defects not only make the microstructures of as-cast NAB more complex but also cause the inhomogeneity of the corrosion product film, resulting in greater stress in the growth process of the film [21]. The increase in growth stress can cause the film to become loose or even crack, thereby causing the corrosive medium to more easily enter the film and corrode the substrate. Finally, the corrosion potential of UNS C95810 decreases due to defects. The lower corrosion potential provides a more powerful driving force for corrosion, thereby accelerating the corrosion of UNS C95810.

### 4.4. Corrosion Mechanism of UNS C95810

**Figure 13:**
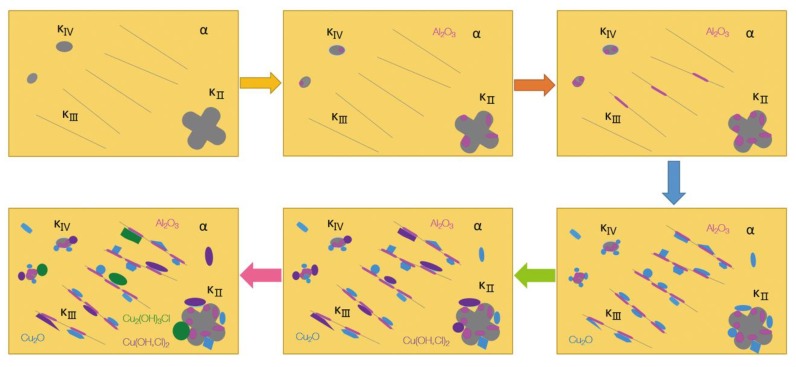
Process mechanism of corrosion behavior of S-Cast immersed in artificial seawater.

According to the experimental results obtained in this paper and related literature [21], the stability of the phases in NAB from low to high is κ_II_, κ_IV_, κ_III_ and. Since the κ phase is an Al-rich phase and Al is a more active metal than Cu, the κ phase will preferentially corrode and form Al_2_O_3_ on the surface when NAB is in contact with seawater. Al_2_O_3_ is a more stable oxide than that of Cu_2_O and CuO, so the κ phase covered by Al_2_O_3_ will no longer corrode as an anode phase but will act as a cathode phase in galvanic corrosion to accelerate the corrosion of the surrounding microstructures. The above results in the formation of Cu_2_O, Cu(OH,Cl)_2_ and Cu_2_(OH)_3_Cl on the surface of the surrounding microstructures. The Volta potential between the κ_II_, κ_III_, κ_IV_ phases and the α phase is 60–80 mV, 10–30 mV and 20–40 mV, respectively [21]. Therefore, the corrosion near the κ_II_ phase is the fastest and the most serious, followed by that near the κ_IV_ phase, and finally that near the κ_III_ phase. In summary, the specific corrosion behavior of S-Cast can be summarized as Figure 13.

**Figure 14:**
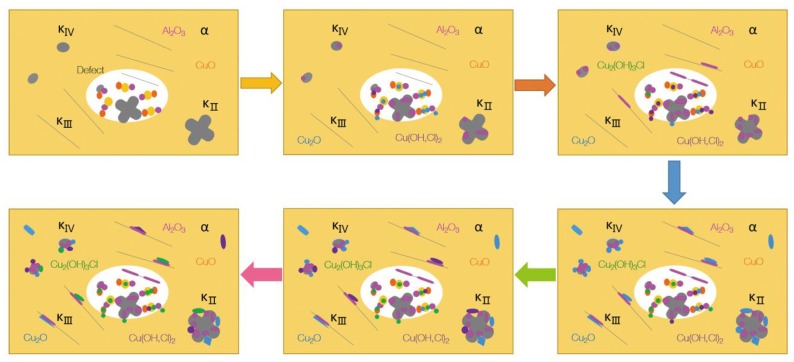
Process mechanism of corrosion behavior of S-Defect immersed in artificial seawater.

For S-Defect, in addition to the corrosion characteristics of S-Cast, it has some additional characteristics due to the presence of defects. The presence of defects not only promotes the formation of galvanic corrosion between the internal microstructures of the defects but also promotes that between the defects and the surrounding microstructures, thereby greatly increasing the tendency toward galvanic corrosion. Therefore, the corrosion behavior of S-Defect can be obtained by combining the corrosion process of S-Cast with an additional galvanic corrosion process introduced due to the existence of defects. Its corrosion mechanism can be summarized as shown in Figure 14.

## 5. Conclusions

In this paper, the effects of cast defects on the corrosion behavior and mechanism of the UNS C95810 in artificial seawater were studied. The conclusions are as follows:

1) The microstructure of the UNS C95810 alloy consists of an α phase and a κ phase. Except for these cast phases, there are oxides of copper and aluminum, along with pure copper in defects.
2) Compared to those of S-Cast, S-Defect has a thicker corrosion product film, more negative corrosion potential and greater corrosion current density.
3) The corrosion product film of both S-Defect and S-cast consists of three layers. The inner layer is Al_2_O_3_ and Cu_2_O with the incorporation of Fe and Ni, the middle layer is Cu(OH,Cl)_2_ or CuO, and the outer layer is Cu_2_(OH)_3_Cl. The defects do not affect the phase composition of the corrosion products, but they have an effect on the amount of the specific phase in the corrosion products.

## Figures and Tables

**Table 1:** Composition of the UNS C95810.

Elements	Cu	Mn	Fe	Al	Ni	Zn, Sn, Pb, C, Si, P, Sb
UNS C95810 (wt.%)	79.9	1.39	4.8	9.24	4.43	Trace

**Table 2:** Chemical components of artificial seawater [18].

Components	NaCl	MgCl_2_	Na_2_SO_4_	CaCl_2_	KCl	NaHCO_3_	KBr	H_3_BO_3_	SrCl_2_	**NaF**
Mass Concentration (g/L)	24.53	5.2	4.09	1.16	0.695	0.201	0.101	0.027	0.025	0.003

**Table 3:** The composition of the studied specimens by SEM with EDS.

Content of Element (atom.%)							
Specimen	O	Al	Si	S	Cl	Fe	Cu
Selected point in Figure 5							100
Selected area in Figure 5	59.29	13.39	4.09	1.68	1.59	1.64	18.32

**Table 4:** The composition of the studied position by SEM with EDS.

Content of Element (atom.%)									
Position	O	Mg	Al	S	Cl	Ca	Fe	Ni	Cu
Zone I in Figure 7e	78.79		2.99	1.30	3.43	0.85	0.85		11.79
Zone II in Figure 7e	72.01	2.31	6.62	1.38	3.67	0.29	0.47	1.13	12.12

**Table 5:** Corrosion parameters of S-Cast and S-Defect.

Sample	Mean (Standard Deviation)		
	Potential vs. SCE (mV)	Current Density (μA⋅cm^−2^)	Rp (kΩ⋅cm^2^)
**S-Cast**	-3.83 (1.93)	0.228 (0.12)	18.9 (2.12)
**S-Defect**	-86.31 (3.62)	0.23 (0.22)	14.9 (2.89)

**Table 6:** The composition of S-Defect immersed in artificial seawater for 3 h.

Content of Element (atom.%)							
Specimen	O	Al	Mn	Cl	Fe	Ni	Cu
Position I	73.30	3.33	-	2.13	2.90	1.44	16.90
Position II	-	15.07	1.41	-	2.65	2.33	78.54
Position III	-	29.27	2.6	-	46.35	12.96	8.82
